# The Biology of the Cattle Tick*Read before the Washington Biological Society, Feb. 3, 1890.

**Published:** 1891-07

**Authors:** Cooper Curtice

**Affiliations:** Veterinarian, Moravia, N. Y.


THE JOURNAL

OF

COMPARATIVE MEDICINE AND

VETERINARY ARCHIVES.

VOL. XII. JULY, 1891. No. 7.

### THE BIOLOGY OF THE CATTLE TICK.*

BY COOPER CURTICE, VETERINARIAN,
MORAVIA, N. Y.

I present to you this evening specimens of the common cattle tick, *Boophilus* (*Ixodes*) *bovis*, Riley, illustrating the successive moults through which the young of this species pass.

The eggs for the experiment in which these ticks were bred were handed me one day last fall by Dr. Theobald Smith. A few adult ticks had been collected from cattle, dropped into an empty bottle, and set aside for a few days. One day he observed that there were also eggs in the bottle and called my attention to them.

On Oct. 10th, I placed some of these eggs in a small glass-covered dish filled with damp mould and set it aside in the incubating room of the laboratory. On Nov. 4th, the young ticks had begun to emerge and by Nov. 15th the hatching was completed, each egg having produced a young tick.

At this time the ticks were taken to the Bureau Experimental Station, and put on a calf which was confined in a stable, whose temperature was maintained at summer heat throughout the experiment. A calf with a white abdomen was selected, thrown on its back, sprinkled with ticks directly on its fine silky hairs, and time allowed for them to crawl into the skin. In this proceeding the certainty of the young ticks arriving at the most suitable surroundings was assured.

* Read before the Washington Biological Society, Feb. 3, 1890.

It is well to state here that the parents of these young ticks were the last seen at the station on any of the cattle, and that the room of experiment and the calf were quite free from ticks before the experiment began. The following table will serve to illustrate the sequence of events in the experiment and present it in rough but compact form.

*Experiment No. 1.—Breeding Ticks.*

| DATE. 1889. | STAGE OF EXPERIMENT. | TIME CONSUMED IN VARIOUS STAGES. |
|---|---|---|
| Oct. 3. | Egg laying begun. | |
| Oct. 10. | Egg laying finished. | Ovipositing one week. |
| Nov. 4. | Ticks appeared. | Hatching three and four weeks. |
| Nov. 15. | Rearing begun. | Unnecessary interval of one week. |
| Nov. 22. | 1st moult, larva to nymph. | Larval stage lasted one week. |
| Nov. 29. | 2nd moult, nymph to adult. | Lasted one week. |
| Dec. 11. | Female ½ grown with male. | About two weeks later. |
| Dec. 16. | Experiment closed. | About one week. |
| | Experiment endured. | About two and one-half months. |

Having briefly reviewed the experiment we may now study its products.

The eggs were laid in a little mass; were subovid, dark brown and opaque and coated with some protective substance. In alcohol they show a thin shell-like covering with a dark opaque mass within. In the latter stages of incubation the form of the young ticks became more and more apparent until they emerged. The exit from the shell seemed to be by the shell rupturing and the imprisoned occupant thrusting it off with its feet. The torn edges afterward rolled inward and produced the appearance of clam shells, so frequently mentioned in writings on this subject.

The anatomical differences of the ticks in their stages will not be discussed further than to point out the gross characters. There are changes which take place in the mouth parts, but these are of such character as to require elaborate drawings and detailed descriptions, and are necessarily omitted.

The young ticks or larvæ when they emerge are whitish, they gradually turn to chestnut brown and resemble minute seeds. Though I have never seen the seed-ticks of the South since I have begun to study them from a standpoint other than to rid myself of them, I think that the young of the cattle tick may form a large portion of them.

The date of the first collection, Nov. 22nd, was a week from the time the young ticks were put on the calf and very fortunately coincided with the time of the first moult. Specimens were taken which showed the tick nearly ready to emerge from the old skin through which they could be seen. The larva (Megnin) is six-footed, possesses no sexual organs and wants the large single stigmata found in later stages. I think it breathes through the three stigmata-like dots I have observed on the side just behind each pair of limbs, but I have not yet made all the sections and examinations necessary to sustain this point. The digestive, urinary tracts, and cloacal pore are present, and apparently vary little in the successive stages.

The next or nymphal stage as seen through the skin of the larva has added a pair of limbs behind the others and a pair of large stigmata behind them. The additional legs lie along the sides in a loop with its convexity directed caudally. The contents of the three front pairs of legs, have withdrawn until only their white tips remain in the test about to be moulted. The head itself is withdrawn from the external skin and the mouth parts seem to be entirely disconnected from it.

Moulting in this stage is completed by the testa first splitting along the side in the vicinity of the head, the feet being thrust out through it, and the dorsal and ventral halves torn apart by the force of the limbs. Specimens of the loose valves, and a tick with its feet thrust through the slit near the head, which are now in the Bureau collection, demonstrate this satisfactorily.

In the change from the larval to the nymphal stage there is a complete ecdysis accompanied by the addition of new parts. The change is, therefore, incomparable to the stage of metamorphosis in insects, and the terms *larva* and *nymph* can only be used in a restricted sense as applied to these stages as being analogous, but not homologous with those stages bearing the same names among the insects. The important changes at this moult occur in the locomotory and respiratory organs.

The next specimens were collected a week later on Nov. 29th. The collection contained individuals about to change from the nymphal to the adult stage which, like the others, could also be seen through the testa they were about to shed. I had previously been enabled to study this stage from field collections, but the specimens collected at this time were invaluable in completing the life-history of the ticks as learned from a single brood.

The moulting at this period is complete. The limbs and head are withdrawn a little from the old testa. The valves split along the sides as in the earlier moult. The digestive and respiratory and locomotory organs undergo little, if any, modifications. The greatest change occurs in the reproductive organs which have developed during the nymphal stage and assume their functions immediately after the ecdysis, when the genital orifices appear in the new skin. Beyond this and the more decided adult characters offered in conformation there seem to be no essential changes.

After this moult the couples mate, and while the male grows but little, if any, afterward, the female continually increases until she reaches the comparatively gigantic size which nearly all are familiar with. They pass through no other moult. The stages passed through by the ticks are therefore four: the egg, the larva, the nymph, the adult. The moults are two: from larva to the nymph, and from the nymph to the adult.

In presenting these few observations on the moultings of the cattle tick I have avoided entering into details on the one hand, and into the broader field of comparative work on the other. The points that I have endeavored to present are:

1st. That cattle ticks can be bred in the laboratory and under comparatively simple surroundings which, during certain seasons of the year, occur everywhere within the cattle tick area.

2nd. That the ability of young ticks to attach to the calf and their subsequent rapid increase in size demonstrates that they may be true parasites whose development can only take place immediately after they become parasitic. Specimens of the same breed reserved in the incubating glass underwent no change, although kept until after the rearing experiment was terminated. Field collections, comprising all stages from larva to adult, also sustain this view.

3rd. That the tick passes through four stages, viz.: 1st, egg; 2nd, larva; 3rd, nymph; and 4th, adult. (*a*) That in the egg the protective, digestive, respiratory, urinary and nervous systems are developed, or those systems which are essential to individual life. (*b*) That at the end of the larval stage there is an addition to the locomotory apparatus and important modifications in the respiratory system, which appear in the nymphal stage. (*c*) That in the nymphal stage the most important changes lie in the development of the generative system, or that, essential for the preservation of the species. (*d*) That in the adult few important changes occur, except those in form which more strongly define the sexes.

The novelty of this communication consists in the presentation of a series of ticks from a single brood, which demonstrates the life cycle of a single species, and of descriptions and drawings of the important stages which have been fairly well outlined by earlier observers from their field collections. These drawings are now in the Bureau collection.

Aside from the purely biological interest excited by the above is the practical one of the relation of the tick to Texas Cattle Fever. Having learned to breed the ticks in quantity I conceived the experiments of taking Northern calves and placing young ticks upon them to determine the extent and kind of injury that the ticks should cause their hosts. These experiments were planned for the summer of 1890, and were sanctioned by Dr. D. E. Salmon, the Chief of the Bureau of Animal Industry, where I was then employed. I, however, was sent to another field, but not to the detriment of the experiment, for I found on my return that they had been carried out on quite a large scale as detailed in the Report of the Secretary of Agriculture for 1890 (p. 109). While Dr. F. L. Kilborne, of the Bureau, had experimentally shown the year preceeding (1889) that ticks had something to do with Southern Cattle Fever, there was, after all, no way of better settling the question than by placing young ticks hatched from the egg directly upon hitherto uninfected cattle. The results of experiments, as carried out by Doctors Smith and Kilborne, showed that Northern cattle purposely infected with cattle ticks afterward presented all the symptoms of Southern Cattle Fever together with the protozoan parasite which Dr. Smith claims to be the cause of the fever.

*Boophilus* (ox loving), is a name I applied as a generic name for these ticks in a paper on "The classification of American Ticks" (illustrated), and read before the Washington Biological Society, December 27, 1890. In the proposed genus the *rostrum* and *palpi* are very short; the *capitulum* is wider than the combined width of the *palpi* and *rostrum;* the second and third segments of the *palpi* are nearly equal and *each widest about the middle where the sides project in an angle;* eyes are present. Koch (in Uebersicht des Arachuidens System, Zecken), seems to have figured this or nearly related species under his genus *Hæmaphysalis*, but so defined the germs that late authorities on the IXODIDÆ include quite other forms of ticks in it. As this species cannot be classified with forms having the *posterior border of the second joint* projected laterally, I have created a new genus.

Prof. C. V Riley, in 1869, Govt. Rept. Diseases of Cattle by

Gamgee, was the first to furnish a good, unmistakable description and figures, excepting the male, of this species. There is a possibility, however, that his description may have been antedated by Koch (1844) who described *Hæmaphysalis rosea*, from Cuba, and by Say (1820) who described *Ixodes annulatus*, from Florida. *Boôphilus bovis* is the only species in this country that I have so far met with to which the characters of *Ixodes annulatus* apply. The above descriptions of Koch and Say are so imperfect that it seemed preferable to adopt the later description.

More lately, Megnin, in his volume on parasites (1883) has described a tick from Algerian cattle as *Ixodes dugesii* which seems to be our *Boophilus bovis*. I have, through the great courtesy of Prof. A. Raillet, of Alfort, received, among other parasites, specimens of ticks from Egyptian cattle. I cannot find a specific difference between the specimens of males and females received from Egypt, and specimens of our *Boophilus bovis*, on direct comparison.

In view of the exact specific identity of our cattle tick with that from Egypt, and the method by which the Southern cattle have been introduced in the early Spanish invasion, and from the fact that the tick completes its development from six-footed stage to adult on cattle, it seems to me that the common cattle tick of the South, *Boophilus bovis*, is an *introduced* species, having been introduced early in the sixteenth century into the Spanish settlements of America. The species thence spread with the cattle into all those parts where the climatic conditions were suitable.

If cattle ticks produce Southern Cattle Fever the bearing of this distribution upon the fever question is seen to be quite important. For South Americans and Algerians, probably, and Egyptians, certainly, having the same fever-producing tick, should have, if the theory of the relation of the tick to Texas fever be correct, the same cattle fever due to the tick.

In the probability that the ticks have been introduced, there is, if the experiments of the Bureau of Animal Industry have been interpreted correctly, also a probability that the Southern Cattle Fever, which also used to be called Spanish Cattle Fever may have also been introduced from the Old World.

The presence of the tick in northern Africa and southern Europe and northern South America, unassociated with a disease similar to our Southern cattle fever, would be very good evidence that the tick was not connected with the well known scourge as exciting cause. Until more is known about the relation of ticks to disease of cattle in those countries few deductions can be made.

It is possible that with an increasing traffic in cattle between Algeria and France we may yet learn whether native French or English cattle may be taken with impunity from a disease similar to Southern Cattle Fever, to Algeria, and whether Algerian cattle may be pastured with Northern cattle with no danger to the latter.

So far as my information extends the traffic has so far extended to the shipment of cattle from Algeria for abattoir purposes alone. The extensive interchange of feeders or stock cattle from one field to another as practiced in this country has apparently had no following on the other side, and hence they have been quite free from any untoward results.

